# Validating a multi-locus metabarcoding approach for characterizing mixed-pollen samples

**DOI:** 10.1186/s13007-023-01097-9

**Published:** 2023-11-04

**Authors:** Sydney B. Wizenberg, Laura R. Newburn, Mateus Pepinelli, Ida M. Conflitti, Rodney T. Richardson, Shelley E. R. Hoover, Robert W. Currie, Pierre Giovenazzo, Amro Zayed

**Affiliations:** 1 Department of Biology, York University, 4700 Keele St., Toronto, ON M3J 1P3 Canada; 2 Appalachian Laboratory, University of Maryland Center for Environmental Science, Frostburg, MD 21613 USA; 3 Department of Biological Sciences, University of Lethbridge, 4401 University Drive W, Lethbridge, AB T1K3M4 Canada; 4 Department of Entomology, University of Manitoba, 12 Dafoe Road, Winnipeg, MB R3T2N2 Canada; 5 Département de Biologie, Université Laval, 2325 Rue de l’Université, Québec City, Québec G1V0A6 Canada

**Keywords:** Pollen, Pollination, Metabarcoding, Metagenetic, Palynology, Melissopalynology

## Abstract

**Background:**

The mutualistic interaction between entomophilous plants and pollinators is fundamental to the structure of most terrestrial ecosystems. The sensitive nature of this relationship has been disrupted by anthropogenic modifications to natural landscapes, warranting development of new methods for exploring this trophic interaction. Characterizing the composition of pollen collected by pollinators, e.g. *Apis mellifera*, is a common means of exploring this relationship, but traditional methods of microscopic pollen assessment are laborious and limited in their scope. The development of pollen metabarcoding as a method of rapidly characterizing the abundance and diversity of pollen within mixed samples presents a new frontier for this type of work, but metabarcoding may have limitations, and validation is warranted before any suite of primers can be confidently used in a research program. We set out to evaluate the utility of an integrative approach, using a set of established primers (ITS2 and rbcL) versus melissopalynological analysis for characterizing 27 mixed-pollen samples from agricultural sites across Canada.

**Results:**

Both individual markers performed well relative to melissopalynology at the family level with decreases in the strength of correlation and linear model fits at the genus level. Integrating data from both markers together via a multi-locus approach provided the best rank-based correlation between metagenetic and melissopalynological data at both the genus (ρ = 0.659; p < 0.001) and family level (ρ = 0.830; p < 0.001). Species accumulation curves indicated that, after controlling for sampling effort, melissopalynological characterization provides similar or higher species richness estimates than either marker. The higher number of plant species discovered via the metabarcoding approach simply reflects the vastly greater sampling effort in comparison to melissopalynology.

**Conclusions:**

Pollen metabarcoding performed well at characterizing the composition of mixed pollen samples relative to a traditional melissopalynological approach. Limitations to the quantitative application of this method can be addressed by adopting a multi-locus approach that integrates information from multiple markers.

**Supplementary Information:**

The online version contains supplementary material available at 10.1186/s13007-023-01097-9.

## Background

Entomophilous pollination, the process by which insects collect and disperse pollen produced by flowering plants, is foundational to the structure of many terrestrial landscapes and success of agricultural systems [1–5]. The mutualistic interaction between producers and primary consumers is beneficial to both parties; entomophilous (insect-dispersed) plants intrinsically rely on insects for successful reproduction—female gametes are sessile, and thus require deposition of male gametophytes by external means [6]. To entice pollinating insects, plants have evolved carbohydrate-dense nectar and nutrient-dense gametophytes, providing a necessary source of food for many pollinating species [7–9]. Due to the sensitive nature of entomophilous (insect-based) pollination, disruptions to the temporal and spatial factors that impact populations of pollinating insects and the plants they feed upon could prove to be detrimental to terrestrial landscapes. Anthropogenic disruption of natural spaces, most prominently seen in the form of habitat fragmentation, biodiversity loss, pollution, and global climate change, could threaten this relationship and induce broader ecological changes via trophic disruptions [10]. To better understand the mutualistic interplay between plants and pollinators, we must first be able to characterize their interaction—a logistically constrained task. Recently, pollen metabarcoding has presented a new frontier for exploring plant–pollinator interactions across environmental gradients [10], but the method is relatively novel and thus requires further validation across a broader range of sampling sites before it can be comprehensively adopted as a means of exploring this vitally important relationship [11].

Agricultural systems are often reliant on insect-based pollination due to the prominence of entomophilous dispersal among angiosperms [12]. Plant species predominantly rely on one means of pollen dispersal, leading to the evolution of specialized gametophytes to maximize pollination success under those conditions [13]. As a result, the two most prominent forms of dispersal are associated with substantial differences in the structure [14] and composition [15] of the vegetative exine of the pollen grain, rendering entomophilous (insect-based) pollen a poor performer of anemophilous (wind-based) pollination, and vice versa. Bearing this in mind, abiotic pollination is unlikely to provide sufficient dispersal in entomophilous species, increasing their reliance on pollinating insects for reproductive success. Native pollinator decline has proved to be detrimental to many wild plant species and crops grown in agricultural spaces [3], leading to the growing reliance on managed western honey bee (*Apis mellifera*) colonies that are strategically placed in close proximity to crops to increase the frequency of pollination events—a multibillion dollar industry [16]. Exploring the factors that drive foraging preference of managed honey bee colonies could provide important insights about their effectiveness as pollinators in natural and agricultural systems, but this goal has previously been difficult to achieve, as observational work is both time and resource intensive. Similarly, studies on foraging ranges and the diversity of food sources in other bee species would benefit from improved mechanisms for identifying pollen species. Pollen metabarcoding presents an opportunity to rapidly characterize the diversity and abundance of pollen collected by bees by identifying the species associated with each pollen grains haplotype [17, 18].

Conceptually similar to DNA barcoding, metabarcoding relies on universal markers associated with conserved regions of the plant genome, wherein high interspecific sequence variation but low intraspecific variation facilitates identification of pollen species [19]. Many markers have been developed for amplifying DNA from pollen grains, but a smaller subset have shown strong promise in their ability to interspecifically differentiate between pollen haplotypes. ITS2, the internal transcribed spacer 2 region of nuclear ribosomal DNA, is a universal marker that has been established for accurate DNA barcoding of both plant and animal species [20]. However, more recent work suggests that its value lies in qualitative identification of species, rather than quantitative estimates of abundance [11, 21]. When compared to microscopic pollen counts, ITS2 provided genus-level sensitivity at identifying the diversity of pollen in a mixed sample, but quantitative measures were not strongly correlated with equivalent melissopalynological estimates [21]. Notably, the conclusion of this work is limited by a small sample size (n = 4) at a single site in Ohio [21]. Contrastingly, the rbcL (ribulose-biphosphate carboxylase large subunit) gene shows promise in its ability to quantitatively identify species in a mixed pollen sample, but with lower taxonomic resolution than ITS2 [19]. Though this work was not constrained by a small sample size, sampling was limited to 13 sites in a single state in the United States of America [19] and thus does not represent the diversity of samples that could be expected from sites across North America. Bearing in mind the limitations associated with each of these markers, a multi-locus approach that integrates information from both ITS2 and rbcL is likely to provide the most accurate characterization of mixed pollen samples [11, 22]. Integrative approaches to metabarcoding are standard practice in other fields [23–26], with most work recommending a combination of primers for characterization, regardless of the study system.

To confidently apply pollen metabarcoding to evolutionary and ecological questions, the approach must first be validated via comparison to melissopalynological data—an endeavour undertaken by Richardson et al. [11]. Their work found that a multi-locus approach can overcome limitations associated with individual primers and reduce the frequency of non-detection and underrepresentation in pollen metagenetic datasets, though their work was conducted at a relatively small geographic scale (6 samples from sites in West-central Ohio) and it is not clear if pollen metabarcoding can generate reliable quantitative data across continental-scale landscapes [11]. Consolidating this information with previous work [21], the utility of ITS2 may be limited to qualitative genus-level detection, whereas rbcL is likely to provide accurate quantitative detection of pollen abundance but may be limited to identification at higher taxonomic levels [19]. Other validation projects have employed a similar method of comparing metagenetic data to melissopalynological estimates, but have often focused on the floral composition of honey, as opposed to fresh pollen collected by foraging bees, or processed pollen balls (i.e. bee bread) stored in colonies [27, 28]. Though previous work on this topic has generally drawn consistent conclusions about the utility of this method, to date no experiment has sought to replicate these findings across a broad range of sampling sites, and a more substantial number of mixed-pollen samples. Building on previous work [11, 21], we set out to test the utility of ITS2 and rbcL for identifying the composition of 27 pollen samples at multiple agricultural sites across Canada. We used melissopalynological data as a point of comparison and asked: does a multi-locus approach, integrating information from both ITS2 and rbcL, provide accurate identification of the abundance and diversity of pollen in mixed samples from honey bee colonies located across Canada?

## Results

### Quantitative characterization

**Fig. 1:**
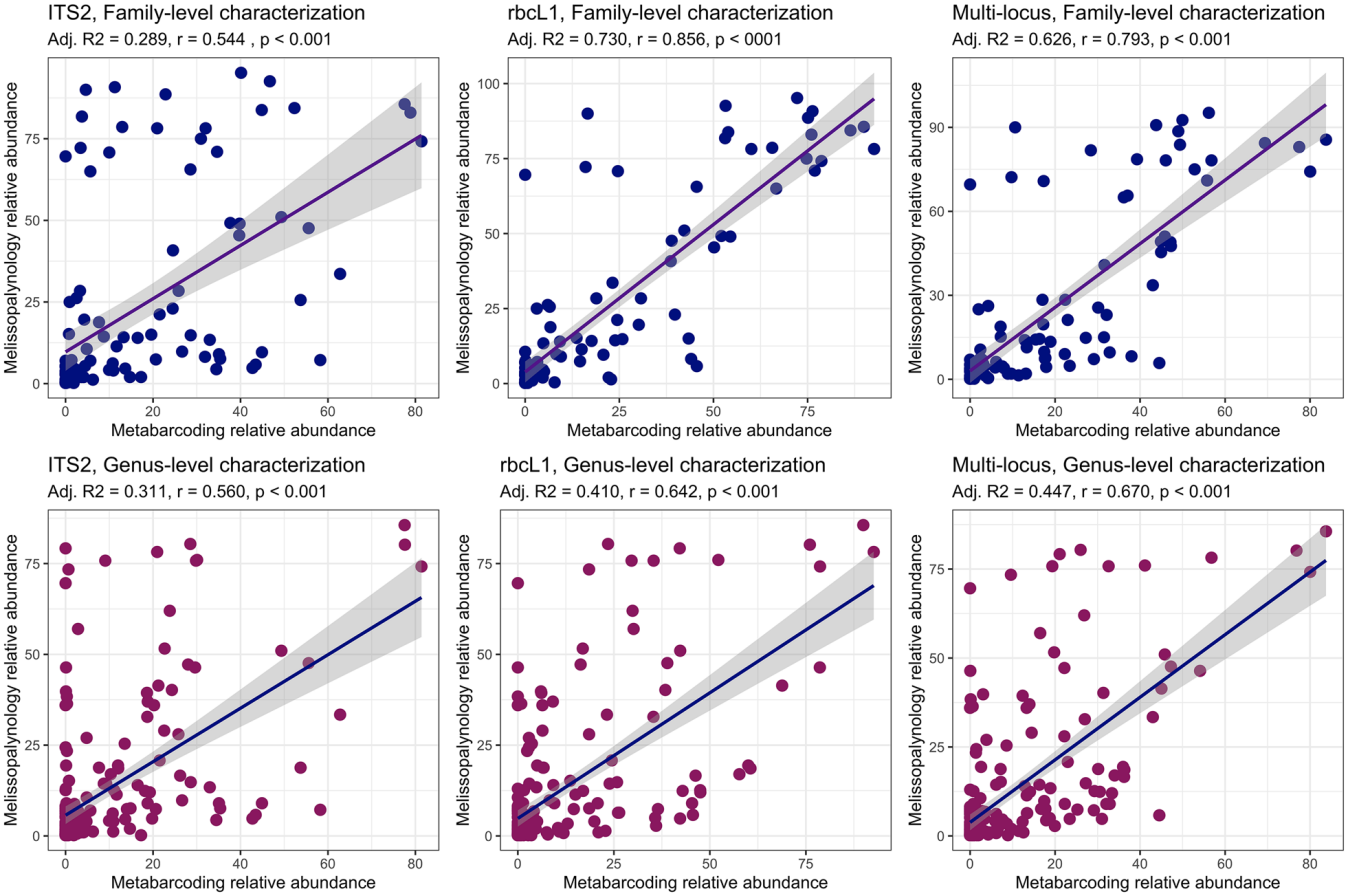
Linear models fitted to predict melissopalynological data from metagenetic data

Pollen metabarcoding data was significantly correlated with melissopalynological data at both the genus and family level (Table 1; Fig. 1). General linear models performed moderately at predicting one value from the other; at the family level, rbcL1 (Adj. R^2^ = 0.730, r = 0.856, p < 0001) substantially outperformed ITS2 (Adj. R^2^ = 0.289, r = 0.544, p < 0.001) at quantitative characterization. Multi-locus averages substantially increased model fit (Adj. R^2^ = 0.626, r = 0.793, p < 0.001) relative to ITS2, but did not outperform rbcL1 at single-locus characterization. At the genus level, multi-locus averages provided the best model fit (Adj. R^2^ = 0.447, r = 0.670, p < 0.001), and rbcL1 (Adj. R^2^ = 0.410, r = 0.642, p < 0.001) again outperformed ITS2 (Adj. R^2^ = 0.311, r = 0.560, p < 0.001) at single-locus characterization. Spearman’s rank-based correlation, which measures the strength of linear association between the relative rank of values (as opposed to raw observations), was high across both markers and taxonomic levels (Table 1). At the family level, multi-locus averages (ρ = 0.830, p < 0.001) outperformed both rbcL1 (ρ = 0.816, p < 0.001) and ITS2 (ρ = 0.724, p < 0.001). Similarly, at the genus level, multi-locus averages (ρ = 0.659, p < 0.001) outperformed both rbcL1 (ρ = 0.611, p < 0.001) and ITS2 (ρ = 0.560, p < 0.001).

### Qualitative characterization

**Fig. 2:**
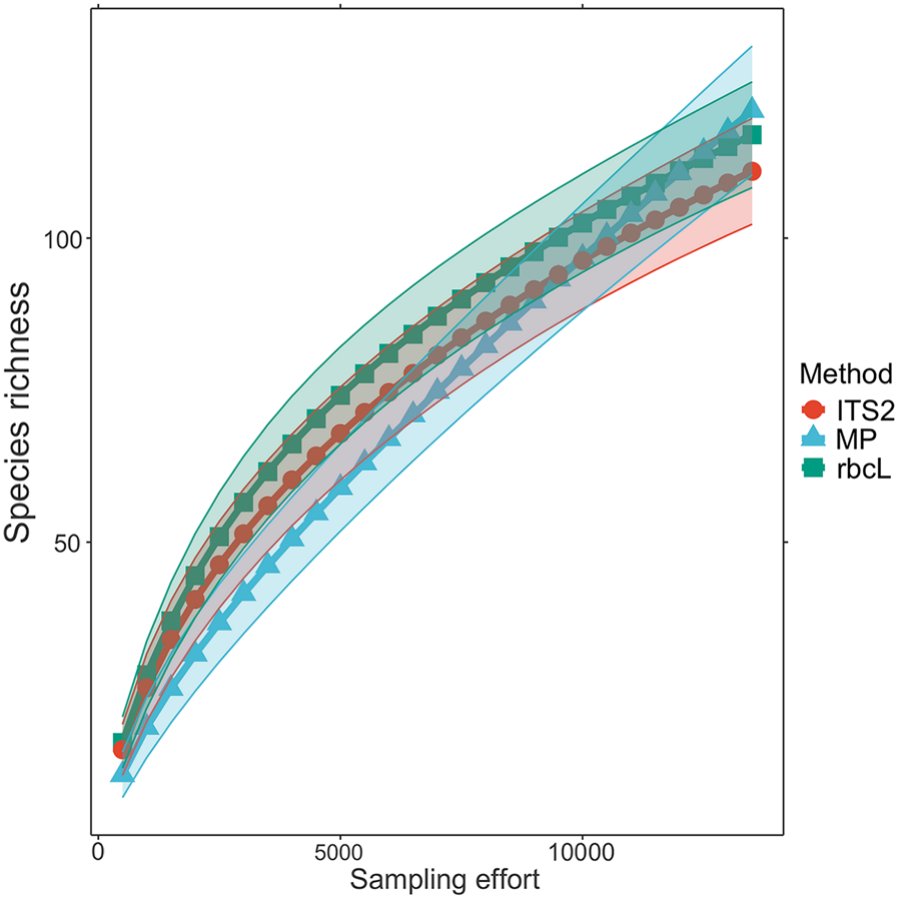
Species accumulation curves as a function of sampling effort for traditional and metabarcoding methods of pollen identification

Qualitative agreement, a measure of the proportion of observations that demonstrated identical presence or absence outcomes across melissopalynological and metagenetic datasets, was high at both the genus and family level. Genus level qualitative agreement was highest for multi-locus averages (77.38%), followed by ITS2 (69.23%) and rbcL1 (66.97%). At the family level, qualitative agreement was again highest for multi-locus averages (82.76%), followed by rbcL1 (76.72%) and ITS2 (75%). Average species richness was high for both ITS2 (28.07 ± 11.19) and rbcL1 (25.67 ± 10.21) relative to melissopalynology (8.19 ± 3.77). This is attributable to the substantial increase in sampling effort, e.g. the size of the pollen pool undergoing assessment, between metabarcoding (ITS2: 97,027 ± 23,684 reads per sample; rbcL: 60,940 ± 18,562 reads per sample) versus melissopalynology (n = 500 grains per sample). Species accumulation curves indicate that after controlling for sampling effort, melissopalynological characterization provides a similar or higher species richness value than either marker (Fig. 2).

### Cost

Melissopalynology, which assessed 500 pollen grains per sample, cost $200/sample ($5400 total for this project), and $0.40 per pollen grain. In contrast, single marker metabarcoding cost, on average, $56.60/sample. Carrying out the analysis with 2 markers, as presented here, costs on average, $98.77/sample (note: the cost of metabarcoding with 2 markers is not exactly double that of a single marker because only a single DNA extraction is needed). Single locus barcoding using ITS2 on average generated 97,027 (± 23,684) reads per sample, equating to a cost of $0.0006 per barcode read. Single locus barcoding using rbcL1 generated an average of 60,940 (± 18,562) reads per sample, equating to a slightly higher cost of $0.0009 per barcode read. Working under the assumption that each pollen grain equates to a single barcode read, both single-locus barcoding and multi-locus barcoding provide a significantly lower assessment cost per pollen grain. Independently of that assumption, assessing a full batch of 84 pollen samples by melissopalynology ($16,800) is significantly more expensive than single locus ($4,754.40) or multi-locus ($8,296.8) metabarcoding. All costs reported above are in Canadian dollars.

**Table 1:** Linear models fitted to predict melissopalynological data from metagenetic data, and related correlation tests

Taxonomic level	Marker	Adj. R^2^	RSE	p-value	Pearson’s r	Spearman’s ρ
Family	ITS2	0.289	25.14	< 0.001	0.544***	0.724***
	rbcL1	0.730	15.49	< 0.001	0.856***	0.816***
	Average	0.626	18.24	< 0.001	0.793***	0.830***
Genus	ITS2	0.311	16.26	< 0.001	0.560***	0.585***
	rbcL1	0.410	15.05	< 0.001	0.642***	0.611***
	Average	0.447	14.57	< 0.001	0.670 ***	0.659***

Linear models fitted to predict melissopalynological proportional data from paired metagenetic proportional data. Adj. R^2^ is the adjusted model fit statistic for each respective linear model, RSE is the residual standard error. Pearson’s r is the product-moment correlation coefficient, Spearman’s ρ is the rank-based correlation coefficient

***Indicates a statistically significant relationship (p < 0.001). Average is the multi-locus average across both primers

Linear models fitted to predict melissopalynological relative abundance values from multi-locus average proportional data at two taxonomic levels (family and genus). Summary statistics for each model are listed above; Adj. R^2^ is the linear model fit, r is Pearson’s product-moment correlation coefficient, and p is the statistical significance of linear association.

Rarefaction curves predicting changes in species richness, the number of unique plant genera detected, as a function of sampling effort. Method refers to the means of assessment, ITS2 and rbcL are each of the respective markers employed in our protocol, MP is melissopalynology, microscopic pollen assessment performed by a taxonomist. Sampling effort represents the number of grains of pollen examined under the microscope for MP, or number of sequencing reads for ITS2 and rbcL. Please note that that MP data was forecasted based on a sample size of 500 grains using the *BiodiversityR* package (v. 2.15, 2023-05-07) [45].

## Discussion

Broadly, pollen metabarcoding data was significantly positively correlated with melissopalynological data across both primers and taxonomic levels (Table 1). At the genus level, multi-locus averages (r = 0.670, ρ = 0.659; p < 0.001) outperformed both rbcL1 (r = 0.642, ρ = 0.611; p < 0.001) and ITS2 (r = 0.560, ρ = 0.585; p < 0.001) via product-moment correlation and rank-based correlation tests. At the family level, rank-based correlation was highest for multi-locus averages (ρ = 0.830; p < 0.001), but product-moment correlation was highest for rbcL1 (r = 0.856; p < 0.001). This suggests that an integrative multi-locus approach is best for quantitative work, but a single-locus approach via rbcL1 can be successfully applied to characterize mixed pollen samples, at least for the Canadian flora captured within our study. When we investigated qualitative agreement across methods, we found that 82.76% of plant families and 77.38% of plant genera present in the melissopalynological dataset were also present in the metagenetic dataset. Thus, qualitative characterization of mixed-pollen samples via presence/absence outcomes across markers shows strong promise in its ability to accurately characterize the composition of mixed-pollen samples. Quantitative characterization, though relatively accurate via a multi-locus approach, is still somewhat limited in its utility.

Our results mirror the conclusions from Richardson et al. [11], who found that multi-locus metabarcoding was more reliable than single-locus metabarcoding. Despite our use of different primers, the general consensus remains true that integrating information from multiple markers provides more accurate characterization of mixed-pollen samples, particularly when evaluating quantitative proportions. Multi-locus averages show strong potential in their ability to overcome limitations associated with metabarcoding when only a single marker is used [11, 22] and thus should be considered standard practice for characterization of mixed-pollen samples. Validation of our multi-locus approach via comparison to melissopalynological analysis has demonstrated the strengths and weaknesses of pollen metabarcoding and has underscored a need to further investigate combinations of markers that provide the most accurate quantitative characterization. Though rank-based correlation was high, a considerable residual standard error and moderate adjusted R^2^ in all linear models demonstrates that this method is imperfect and is prone to some degree of disagreement relative to microscopic pollen assessment.

Disagreement between melissopalynological data and metagenetic characterization could be due in part to the size of samples selected for analysis. Honey bees (*Apis mellifera*) are productive pollinators—they are the most frequent floral visitor of crops around the world [29], and thus are likely to collect pollen in large quantities, particularly when assessed at the colony level by pooling the contributions from many individuals. Both melissopalynological and metagenetic characterization rely on analyzing a smaller subset of pollen, which could lead to over and under-representation of mixed pollen assemblages via either method. Our 27 pollen samples had a variable number of metagenetic hits (e.g., the number of individual reads with a barcoding match) at the genus level but averaged 97,027 (± 23,684) for ITS2 and 60,940 (± 18,562) for rbcL; potentially representing a larger proportion of the pollen pool than the 500 pollen grains assessed via microscopy. Increasing the number of pollen grains that undergo microscopic assessment could improve the confidence surrounding these estimates, but microscopic pollen assessment is a time-consuming and specialized skill, limiting its utility for large-scale pollen assessment and further highlighting the need for modern approaches [17]. Moreover, even expert taxonomists are unable to fully identify all of the pollen grains found in a mixed-pollen sample. For example, on average, our microscopic analysis could not provide a positive identification for 6.09% of the pollen examined, and in one case (sample #9), 44% of the sample was unidentifiable and/or undifferentiable by the melissopalynologist. Based on the taxonomist’s notes, this equated to an estimated 69 unique pollen species that could not be confidently assigned a plant genera.

In addition to rapidly characterizing a larger proportion of the pollen pool, metabarcoding has shown promise in its ability to detect rare plant species [30–33] that often exist in low abundance and thus are prone to misrepresentation when assessed via melissopalynology. On average, metabarcoding detected substantially more genera (ITS2: 28.07 ± 11.19; rbcL: 25.67 ± 10.21) than melissopalynology (8.19 ± 3.77), these inconsistencies could indicate the presence of false positives in the metagenetic datasets, or could be a result of the differences in sample size discussed above. Though false positives remain a concern with metagenetic work in any context, our rarefaction curves (Fig. 2) and high degree of qualitative agreement suggest that our metabarcoding protocol minimizes the risk of erroneous inferences. This is likely attributable to our multi-step quality control (see “Quality control”) that ensure that our final metagenetic datasets are high-quality. Its arguably more plausible that the increased number of taxa detected with metabarcoding are consistent with the greater sampling efforts (examining thousands of reads per sample versus examining 500 pollen grains per sample) as borne out by our rarefaction analysis. Bearing this in mind, metabarcoding could provide more accurate estimates of sample composition with lower standard errors at a considerably cheaper cost, relative to melissopalynological assessment.

Melissopalynological characterization may be more robust in its ability to identify the precise number of pollen grains belonging to a specific species in a mixed-pollen sample but suffers from its own set of limitations. Microscopic identification of pollen grains generally relies on size and exine morphology to distinguish between species [17, 34]; however, closely related taxa often feature similarities in their appearance [35] and therefore may be difficult to differentiate without genetic analysis. This issue is further highlighted by *A. mellifera*’s preference for entomophilous species; pollen grains evolved for specialized dispersal often feature similar characteristics, such as the composition and structure of the vegetative cell walls [13, 35]. Pollen metabarcoding overcomes this limitation by identifying closely related species through differential gene sequences, based on the foundational assumption that intraspecific variation is less than interspecific variation [18, 36]. This assumption is critical to the development of primers for metabarcoding, but issues can arise when related species display low (or no) sequence divergence, and thus insufficient interspecific variation for differentiation [11]. Integrating information from multiple markers for a multi-locus approach is one means of overcoming this limitation and can increase the degree of confidence surrounding quantitative characterization of mixed-pollen samples as demonstrated above. Bearing this is mind, future work should rely on a multi-locus approach and should focus on increasing the accuracy of quantitative characterizations by developing additional primers and testing their utility independently and in conjunction with established primers.

## Conclusions

Integrating information from two primers to generate multi-locus average relative pollen abundance values increases the accuracy of pollen metabarcoding as a method of analyzing the composition of mixed-pollen samples. Multi-locus characterization was more strongly associated with melissopalynological data than values generated by either primer individually, highlighting a need to employ multiple markers in any study utilizing this method. Though rank-based and product-moment correlation between metabarcoding and melissopalynological data was high, the associated linear models showed a modest model fit and a large residual standard error that increases proportionally with abundance values. Bearing this in mind, characterization using multi-locus average relative pollen abundance values shows strong methodological utility for understanding the trends (as opposed to individual quantitative values) surrounding the composition of mixed-pollen samples. Though metabarcoding-based characterization may carry a lower degree of confidence relative to melissopalynological methods (but see caveats about limitations of melissopalynological data in the discussion) it is inherently more amenable to high-throughput analyses at a scale that is previously unfathomable for melissopalynological research. Finally, we caution about the use of a single gene to draw conclusions about the composition of mixed-pollen samples as the multi-locus approach is demonstrably better.

## Methods

To test the utility of pollen metabarcoding for characterizing mixed-pollen samples, we performed paired sample analyses. Each pollen sample underwent both melissopalynological and genetic analysis using two barcoding regions: ITS2 [21, 37, 38], and rbcL1 [39]. The forward and reverse primers used for both PCR1 and PCR2 are reported in Table 2.

**Table 2:** PCR1 and PCR2 primer specifications

Primer	Sequence
PCR1	
rbcL1 (F)	AGACCTWTTTGAAGAAGGTTCWGT
rbcL1 (R)	TCGCATGTACCTGCAGTAGC
ITS2 (F)	ATGCGATACTTGGTGTGAAT
ITS2 (R)	TCCTCCGCTTATTGATATGC
PCR2	
rbcL1 (F)	**CAGCGTCAGATGTGTATAAGAGACAG**AGACCTWTTTGAAGAAGGTTCWGT
rbcL1 (R)	**GCTCGGAGATGTGTATAAGAGACAG**TCGCATGTACCTGCAGTAGC
ITS2 (F)	**CAGCGTCAGATGTGTATAAGAGACAG**ATGCGATACTTGGTGTGAAT
ITS2 (R)	**GCTCGGAGATGTGTATAAGAGACAG**TCCTCCGCTTATTGATATGC

(F) indicates a forward primer and (R) indicates the paired reverse primer. Bolded values are changes to the forward and reverse primers between PCR1 and PCR2

### Sample collection: Manitoba (1–7)

We collected pollen samples from pollen traps (modified OAC Pollen Trap, Ontario Agricultural College, Guelph, ON, Canada) installed at the entrance of honey bee hives at seven sites in southern Manitoba: Glenlea (49.64542443, − 97.1215731), Charleswood (49.8366079, − 97.2969661), Winnipeg site one (49.814012304, − 97.1188160), Richer (49.6917590, − 96.4697017), Haddashville (49.628003, − 95.9189587), Winnipeg site two (49.81401230, − 97.1188160), and Winnipeg site three (49.8089928, − 97.1275037). For the first two samples (Glenlea and Charleswood) the pollen traps were installed on August 2nd, 2019, and the resulting pollen samples were collected on August 6th, 2019. For the third sample (Winnipeg site one), the pollen trap was installed on August 6th, 2019, and the resulting pollen samples were collected on August 9th, 2019. For the fourth and fifth samples (Richer and Haddashville), the pollen traps were installed on July 19th, 2021, and the resulting pollen samples were collected on August 1st, 2021. For the final two samples (Winnipeg sites two and three), the pollen traps were installed on June 27th, 2022, and the resulting pollen samples were collected on July 4th, 2022. We weighed each of the resulting seven pollen samples, which averaged 35.64 g (± 4.19), then sealed and shipped them on dry ice to undergo analysis.

### Sample collection: Alberta (8–13)

We collected pollen samples from pollen traps (Wooden Pollen Traps, Dancing Bee Equipment, Winnipeg, MB, Canada) installed at the entrance of honey bee hives at one site in southern Alberta: Earl’s Forest Yard on the University of Lethbridge main campus (49.67169, − 112.86041). We collected pollen from the traps twice weekly from April 2nd, 2021–October 1st, 2021 and froze the samples at − 20 °C within an hour of being collected. We collected samples from two sets of four hives on an alternating basis (one collection per week from each set of four) and pooled the resulting pollen from both sets of hives on a monthly basis. We collected samples from the combined pollen pool for each of the 6 months during which the experiment took place; each sample represented pollen collected by the honey bees from all eight hives during that month. Sample collection occurred at the end of each month, from April 2021–October 2021, resulting in 6 representative pollen pools (April, May, June, July, August, and September). We weighed 25 g from each of the resulting six samples, then sealed and shipped them on dry ice to undergo analysis.

### Sample collection: Quebec (14–19)

We collected pollen samples from pollen traps (Front Wooden Pollen Trap, Propolis-etc Material Apicole, Montreal, QC, Canada) installed at the entrance of honey bee hives at six sites in southern Quebec: Deschambault (46.6763271, − 71.9180858), Donnacona (46.6901873, − 71.7136567), Neuville one (46.7039023, − 71.6164938), Neuville two (46.7187134, − 71.5527606), Pont-Rouge one (46.7499122, − 71.635718), and Pont-Rouge two (46.7773401, − 71.6438764). We installed the traps on July 18th, 2022, and collected the pollen samples from each trap on July 20th, 2022. We weighed each of the resulting six pollen samples, which averaged 26.9 (± 4.8) g, then sealed and shipped them on dry ice to undergo analysis.

### Sample collection: Ontario (20–27)

We collected pollen samples from pollen traps (Pollen Trap Bottom Board, model #APH1000, ApiHex Beekeeping Supplies, Guelph, ON, Canada) installed at the entrance of honey bee hives at one site in southern Ontario: the Toronto Regional Conservation Authority’s apiary (43.82610980, − 79.6043184). We installed the traps on August 8th, 2022, and collected the pollen samples from each trap on August 15th, 2022. We weighed each of the resulting seven samples of pollen to 20 g, then sealed and shipped them on dry ice to undergo analysis.

### Melissopalynology

Melissopalynological assessment was completed by Johanne Parent at H2Lab in Rimouski, Quebec. The methods reported below detail her procedure, as described to the authors, in first person language. Pollen samples were stored in the freezer at − 80 °C until they underwent analysis. We brought them to ambient room temperature (21 °C) before weighing them. We then measured out 2 g of pollen (1995–2004 g) in a 50 mL centrifuge tube (CELLSTAR 50 mL tubes, Greiner Bio-one, Monroe, NC, USA), added 40 mL of distilled water, sealed the tube, and shook the suspension by hand to encourage dissolution. After allowing dissolution, we vortexed (Vortex Mixer, Fisher Scientific, Waltham, MA, USA) the sample for two minutes to create a homogenous suspension and pipetted a small drop (< 1 mL) onto a glass slide. To improve our ability to see and identify distinct pollen morphology, we used basic fuchsin to stain the vegetative cell. To do this, we added a 2 mm × 2 mm cube of glycerine jelly containing 0.1% basic fuchsin to the slide, then heated it on a histology plate (Otto C. Watzka & Co Ltd., Montreal, QQ, Canada) set to 65 °C, until the jelly had completely dissolved and the preparation was homogenous. We then applied a 22 mm × 22 mm glass cover slide, and sealed it with paraffin, before allowing the slide and fuchsin stain to set at room temperature (21 °C). Once the slide had set, we used a ruler to draw a vertical line directly through the centre of the preparation and performed horizontal transects at 1000× magnification under a light microscope. Transects extended from the centre to the right boundary of the slide cover, starting at the top of the vertical axis, until 500 pollen grains had been assessed and identified.

### Pollen metabarcoding

Pollen samples were stored in the freezer at − 80 °C until they underwent analysis. We brought them to ambient room temperature (21 °C) before starting DNA extractions, using the NucleoMag DNA Food Kit (Macherey-Nagel, Düren, Germany). We started the extraction protocol by combining 10 g of pollen with 20 mL of lysis buffer: 70% autoclaved filtered water (Millipore Sigma, Burlington MA, USA), 20% 10× STE (100 mM NaCl, 10 mM Tris, 25 mM EDTA), and 10% diluted SDS (10% sodium dodecyl sulfate). After combining the buffer and pollen we sealed each sample, inverted it 10 times to promote mixing, then homogenized it into a suspension by mixing each sample for 10 min in an orbital shaker (G25 Incubator Shaker, New Brunswick Scientific, Edison, NJ, USA) at 25 °C and 375 rpm. Immediately after removing the samples from the orbital shaker, we pipetted 3 mL of the homogenized sample into a bead beater tube (Bead Mill 24, Fisherbrand, Ottawa, Ont., Canada) and bead-beat it for 2 min (4 × 30 s. cycle), then placed the sample on ice for 5 min to allow it to cool. After the sample had cooled, we pipetted 550 µL of the homogenized suspension to a 1.5 mL Eppendorf tube. We warmed CF lysis buffer in a 65 °C water bath for 10 min then added 550 µL of the warmed buffer and 10 µL of Proteinase K (from the NucleoMag DNA Food kit) to the Eppendorf tube containing the homogenized sample and vortexed (Mini Vortex Mixer, VWR, Mississauga, Ont., Canada) the sealed tube for 30 s. We then incubated the sample for 30 min at 65 °C in a block heater (Isotemp 145D, 250 V, Fisherbrand, Ottawa, Ont., Canada), inverting every 10 min. After incubation, we added 20 µL of RNase A (New England Biolabs, Ipswich, MA, USA) and allowed the sample to incubate for another 30 min, at room temperature (20 °C). After incubation, we centrifuged the sample for 20 min at 14,000 rpm (Centrifuge 5810 R, 15 amps, Eppendorf, Hamburg, Germany), transferred 400 µL of the upper liquid layer to the binding plate, added 25 µL of NucleoMag B-Beads and 600uL of binding buffer CB (both from the NucleoMag DNA Food kit) then ran an extraction program (KingFisher Flex, ThermoScientific, Waltham, MA, USA). Each of the 5 deep well plates used to complete the extraction program contained either 600 µL of CMW buffer (wash 1), 600 µL of CQW buffer (wash 2.1), 600 µL of 80% EtOH (wash 2.2), or 100 µL of buffer CE (elution). After the extraction program was complete, we transferred 80 µL of the eluted sample to a fresh 1.5 mL Eppendorf tube and froze it at − 20 °C until we began DNA amplification.

PCR was split into two programs: the first of which amplified the region of interest, and the second of which extended the length of the amplified sequence. We used two primers, the forward and reverse sequences for each primer and each PCR program are reported in Table 2. For each PCR program we used 96 well plates, containing 84 pollen samples, 6 negative controls, and 6 positive controls (Banana, *Musa* sp.). We pipetted 11 µL of sterile water, 12.5 µL of 2× Taq Pol Mix (New England Biolabs, Ipswich, MA, USA), 0.5 µL of each relevant forward and reverse primer, and 0.5 µL of sample DNA into each well. We then ran a PCR cycle (Eppendorf Mastercycler, Ep Gradient, Hamberg, Germany) using program specifications indicated below (Table 3). PCR1 product was used as the template for PCR2; the same master mix described above was combined with 0.5 µL of PCR1 product. After each respective PCR program, we used gel electrophoresis to confirm sufficient amplification of each sample and identify any potential contamination using the negative controls. Following PCR2, we prepared samples for Illumina Sequencing by performing a third PCR program that tagged each sample with a unique combination of forward and reverse primers; PCR3 program specifications follow that reported in Table 3, with a primer annealing temperature of 60 °C. We then normalized the resulting PCR3 product using a SequalPrep Normalization kit (Invitrogen, Burlington, Ont., Canada), and shipped the normalized samples on dry ice for Illumina Sequencing (Illumina MiSeq PE250) at Genome Quebec.

**Table 3:** PCR1 and PCR2 program specifications

Program	Step	Temperature ( °C)	Time	Number of cycles
1	Initial denaturation	94	10 min	1×
	Denaturation	94	30 s	40×
	Primer annealing	**54**	40 s	
	Extension	72	1 min	
	Final extension	72	10 min	1×
2	Initial denaturation	94	10 min	1×
	Denaturation	94	30 s	40×
	Primer annealing	**56**	40 s	
	Extension	72	1 min	
	Final extension	72	10 min	1×

Bolded values are changes in annealing temperature between programs. PCR3 followed the program specifications indicated above, with an annealing temperature of 60 °C

### Data processing

All data processing was completed in Python (v. 3.10.7), and R (v. 4.2.1), using the *dada2* (v. 1.16.0, 2020-04-07) [40] and *purrr* (v. 0.3.4, 2020-04-16) [41] packages. We processed returned sequence data by first trimming primers, pairing forward and reverse reads from each sample, filtering out low quality reads and sequencing errors, then grouping identical sequences under unique ASV’s (amplicon sequence variants). We trimmed 20 bp off of the start (or end) of each respective forward and reverse read, using the *dada2* filterandTrim() function, and estimated error rates (to account for sequencing errors) using the *dada2* learnErrors() function. We then aligned the forward and reverse reads using the *dada2* mergePairs() function and removed misaligned sequences and chimeras via the *dada2* removeBimeraDenovo() function. To prepare the resulting sequence data for barcoding assignments, we then performed quality control to ensure that no misaligned sequences or chimeras remained, and formatted the resulting output into a matrix with abundance values for each ASV and sample. We then built a database that linked species to unique sequences associated with each primer using the MetaCurator method developed by Richardson et al. [42]. A detailed description of MetaCurator is provided by Richardson et al. [42]; briefly, MetaCurator uses NCBI reference sequences to curate barcoding databases that link ASV’s to taxonomic assignments, at the species level, accounting for dereplication of identical sequences for taxonomically conserved markers such as *rbcL*. The code we used to generate MetaCurator libraries is publicly available [42].

We used our MetaCurator libraries to parse through returned sequence data and identify the species associated with each ASV, setting a precursory condition of > 0.95 similarity. After identifying the plant species associated with each sequence, we consolidated classifications at two taxonomic levels (genus, and family) and prepared data for filtering. To control for mistagging during sequencing, we utilized a filtering method developed by Richardson [43]. We used negative controls as indicators of mistagging frequency and filtered real sample data to remove detections with a high likelihood of representing mistag-associated false detections [43]. To improve our ability to identify and filter mistagged sequences, we opted to include our 6 positive controls in the filtering program by removing observations of our control substance (*Musa* sp.) and treating them as an additional 6 negative controls.

### Data analysis

All data analysis was completed in R (v. 4.2.1). We first converted all values to proportions of the total sample to control for differences in sample size (e.g., the 500 pollen grains assessed via melissopalynology vs. the number of reads associated with each sample via metabarcoding), and thus total relative abundance. As a result, all values analyzed and presented in the result section are in reference to the relative percentile that each species represented for each respective pollen sample. We paired the observations by consolidating the three datasets based on the species composition of the samples that underwent melissopalynological assessment, extracting and pairing the equivalent sample and species abundance from each respective primer. We did this at two taxonomic levels (genera and family), resulting in two distinct datasets containing the relative proportion of each pollen species, in each sample, for four measures: melissopalynological values, ITS2 metabarcoding values, rbcL metabarcoding values, and the multi-locus average across both primers. The decision to use melissopalynological data as our basis of comparison was a result of the sample size discrepancies between each method of analysis; observations of zero in the melissopalynological dataset were deemed null due to the logistical limitations of melissopalynological assessment. On average, the observations included in analysis represented 71.36% (± 18.07) of the total metagenetic hits across both primers. We first analyzed the data using general linear models, employing the lm() base function included in the base R *stats* package (v. 4.2.1), and used the adjusted R^2^ value to gauge model strength. We plotted the corresponding linear models using the base ggplot() function in the *ggplot2* package (v. 3.3.6, 2023-04-03) [44]. We then evaluated rank-based correlations using Spearmen’s method, via the cor.test() function included in the *stats* package, and product-moment correlations using Pearson’s method, via the cor.test() function included in the *stats* package. We next explored how species richness estimates, the number of unique plant genera identified in each sample, varied between primers and melissopalynology. We did this by generating species accumulation curves, using the accum.long2() function included in the *BiodiversityR* package (v. 2.15, 2023-05-07) [45] and plotting the resulting rarefaction curves in *ggplot2*. The melissopalynologist on average could not confidently identify the species associated with 6.09% of pollen grains that underwent assessment. This equated to 69 unique unidentifiable species, which were denoted as “unknown” genera for generating rarefaction curves. In addition to quantitative analysis, we evaluated the qualitative value of pollen metabarcoding by converting relative proportions to binary presence/absence values and generating the rate of agreement between each of the three methods.

### Quality control

To ensure that our metagenetic data was high-quality we incorporated multiple stages of quality control into both our wetlab and bioinformatics pipelines. We used both positive and negative controls to ensure that (1) our barcoding approach could successfully identify our positive control species (banana, *Musa* sp.) and (2) to ensure that no contamination had occurred during any PCR programs. We filtered out chimeras, bimeras, and misaligned sequences using the removeBimeraDenovo() function in dada2 during data processing. All resulting ASV’s were a minimum of 220 bp in length, and thus did not require additional filtering based on fragment length. We accounted for sequence mistagging, e.g. the erroneous misassignment of a sequence to an incorrect index, using an additional filtering program developed by Richardson [43].

### Cost analysis

To compare the relative cost of melissopalynology, single locus barcoding, and multi-locus barcoding, we approximated the total cost per sample. This included reagents, lab supplies, and the hourly cost of a molecular palynologist. This supply cost estimates are based on recent purchases of lab supplies and reagents, which may vary across regions, in Canadian dollars. DNA extraction on a full batch of 84 samples averaged $420 in supplies and $792 in labour. This cost includes the purchasing of a DNA extraction kit, RNaseA, pipette tips, conical tubes, and KingFisher extraction program plates. Metabarcoding using a single marker averaged $252 in supplies, $1214.40 in labour, and $2076 for sequencing (note: in-house library preparation reduces the cost of sequencing but increases the cost of supplies and labour). This cost includes the purchasing of reagents, PCR plates and pipette tips. Across the entire protocol, single locus barcoding averaged $4754.4, and multi-locus barcoding averaged $8296.8, per 84 pollen samples.

### Supplementary Information

**Additional file 1.** The dataset supporting the conclusions of this article. Genera/family refers to the relevant taxonomic classification at that level. MP% is the relative abundance as assessed via meliossopalynology. ITS2% and rbcL% are the relative abundance as assessed via each respective marker. Avrg% is the multi-locus average relative abundance.

## Data Availability

The dataset supporting the conclusions of this article is included as a Additional file 1.
